# Improving Tree Seedling Quality Using Humates Combined with Bacteria to Address Decarbonization Challenges through Forest Restoration

**DOI:** 10.3390/plants13111452

**Published:** 2024-05-23

**Authors:** Aleksey Nazarov, Sergey Chetverikov, Maxim Timergalin, Ruslan Ivanov, Nadezhda Ryazanova, Zinnur Shigapov, Iren Tuktarova, Ruslan Urazgildin, Guzel Kudoyarova

**Affiliations:** 1 Department of Environment and Rational Use of Natural Resources, Faculty of Business Ecosystem and Creative Technologies, Ufa State Petroleum Technological University, ul. Kosmonavtov 1, 450064 Ufa, Russia; ivanovirs@mail.ru (R.I.); nad-ryazanova@mail.ru (N.R.); umrko@mail.ru (I.T.); urv@anrb.ru (R.U.); guzel@anrb.ru (G.K.); 2 Ufa Institute of Biology, Ufa Federal Research Centre, Russian Academy of Sciences, Prospekt Oktyabrya 69, 450054 Ufa, Russia; che-kov@mail.ru (S.C.); timermax@mail.ru (M.T.); 3 South Ural Botanical Garden-Institute, Ufa Federal Research Center, Russian Academy of Sciences, 450080 Ufa, Russia; shigapov@anrb.ru

**Keywords:** humic substances, seedling growth, rhizosphere microorganisms, decarbonization, forest restoration, *Pseudomonas* sp.

## Abstract

Improving the quality of tree planting material for carbon sequestration through reforestation can help solve environmental problems, including the need to reduce the concentration of carbon dioxide in the atmosphere. The purpose of this study was to investigate the possibility of using humic substances in combination with rhizosphere microorganisms *Pseudomonas protegens* DA1.2 and *Pseudomonas* sp. 4CH as a means to stimulate the growth of seedlings of pine, poplar, large-leaved linden, red oak, horse chestnut, and rowan. Humic substances stimulated the growth of shoots and roots of pine, large-leaved linden, and horse chestnut seedlings. The effects of bacteria depended on both plant and bacteria species: *Pseudomonas protegens* DA1.2 showed a higher stimulatory effect than *Pseudomonas* sp. 4CH on pine and linden, and *Pseudomonas* sp. 4CH was more effective in the case of chestnut. An additive effect of humates and *Pseudomonas protegens* DA1.2 on the growth rate of pine and linden saplings was discovered. Poplar, red oak, and rowan seedlings were unresponsive to the treatments. The growth-stimulating effects of the treatments are discussed in connection with the changes in carbon, chlorophyll, and nitrogen contents in plants. The results show the need for further research in bacterial species capable of stimulating the growth of plant species that were unresponsive in the present experiments.

## 1. Introduction

Humic substances (HSs), which are products of degradation of organic matter contained in soil and extracted from brown coal, peat, and other caustobiolites, are characterized as universal regulators that can improve the properties of both the environment and living organisms [1,2,3]. The present investigation studied the possibility of their use in combination with rhizosphere microorganisms as a means of improving the quality of tree seedlings for forest restoration and solving the problem of decarbonization. It is widely accepted that increasing greenhouse gas emissions are causing global warming and associated climate disruptions, such as frequent droughts and other adverse weather events and natural disasters, triggering increased interest in finding ways to reduce the concentration of carbon dioxide in the atmosphere [4,5]. Growing trees absorb carbon dioxide through photosynthesis and store carbon in their biomass and soils; therefore, large-scale tree planting is seen as an effective way to decrease the buildup of carbon dioxide in the atmosphere [6]. To reduce the concentration of carbon dioxide in the atmosphere, it is important to plant trees not only in forests, but also in urban areas; according to some data, urban areas account for more than half of carbon dioxide emissions [4]. At the same time, according to American researchers, large-scale achievement of global reforestation goals, aiming to mitigate the effects of climate change, will require an increase in the number of tree seedlings by 2–3 times, compared with their number in existing nurseries [7].

The quality of tree seedlings plays an important role in determining the productivity of nurseries [8]. To increase the productivity of tree nurseries, it is promising to use preparations of humic substances and bacteria when growing tree seedlings [9]. It is well known that rhizosphere bacteria [10,11,12], as well as humic substances [13,14,15], stimulate plant growth when used individually. Although much less is known about their effect on trees than on herbaceous plants, it has been found that both humates [16,17] and rhizosphere bacteria [18,19] enhance the growth of woody plants. However, only a few studies have addressed the simultaneous application of these biostimulants. Previously, we successfully used a combination of humates and rhizosphere bacteria from the *Pseudomonas* genus to accelerate the growth of pine and, albeit less successfully, poplar seedlings [9]. However, it is important to test the effectiveness of this treatment on seedlings of a wider range of tree species. Species diversity provides the basis for adaptation and stress tolerance, which is necessary for the long-term survival of forests [20]. It increases the likelihood that at least some individuals of a plant species will be able to adapt to changing environmental conditions.

In connection with the above, the purpose of this project was to study the effect of treating seedlings of not only Scots pine and poplar, but large-leaved linden, red oak, horse chestnut, and rowan, with a combined preparation of humates and strains of rhizosphere bacteria *Pseudomonas protegens* DA1.2 and *Pseudomonas* sp. 4CH. Seedling growth and carbon contents in some of them were followed. In addition, the objective of the research was to study the effect of these treatments on pine seedlings that are lagging in growth. Such seedlings are usually discarded, thereby limiting production of planting material. We hypothesized that treatment with humates and *Pseudomonas* strains could stimulate the growth of undersized seedlings. Thus, the task of this work was to study the impact of species diversity of woody plants in the process of developing a complex preparation of humates and rhizosphere bacteria to improve the quality of seedlings for reforestation and to increase carbon sequestration.

## 2. Results

### 2.1. Shoot Elongation and Accumulation of Seedling Biomass

**Figure 1:**
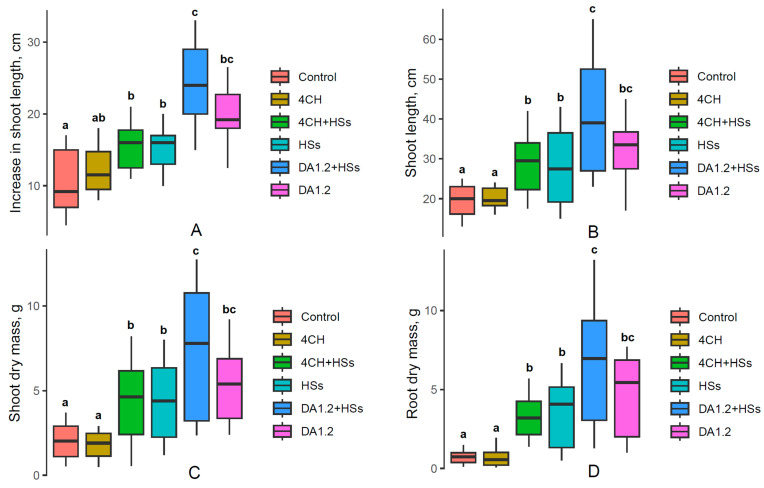
Increase in shoot length during 2 months of growth (average values for the main and lateral shoots) (**A**), shoot length at the end of the growing season (**B**), and shoot (**C**) and root (**D**) dry mass of 2-year-old large-leaved linden (*Tília platyphýllos*) seedlings (n = 10) after their treatment with bacterial preparation (*Pseudomonas* sp. (4CH) and *Pseudomonas protegens* (DA1.2)) as well as humic substances (HSs), used separately or combined with each other and without treatment (control). Boxplots show medians and distributions of values. The whiskers indicate the minimum and maximum values. Statistically different means are marked with different letters, *p* ≤ 0.05 (one-way ANOVA combined with Duncan’s test).

**Figure 2:**
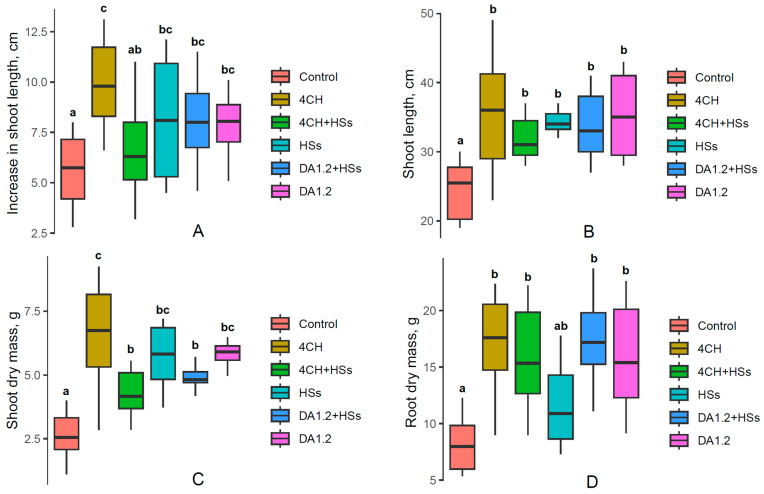
Increase in shoot length during 2 months of growth (average values for the main and lateral shoots) (**A**), shoot length at the end of the growing season (**B**), and shoot (**C**) and root (**D**) dry mass of 2-year-old horse chestnut (*Aesculus hippocastanum*) seedlings after their treatment with bacterial preparation (*Pseudomonas* sp. (4CH) and *Pseudomonas protegens* (DA1.2)) as well as humic substances (HSs), used separately or combined with each other and without treatment (control). Boxplots show medians and distributions of values. The whiskers indicate the minimum and maximum values. Statistically different means (n = 10) are marked with different letters, *p* ≤ 0.05 (one-way ANOVA combined with Duncan’s test).

**Figure 3:**
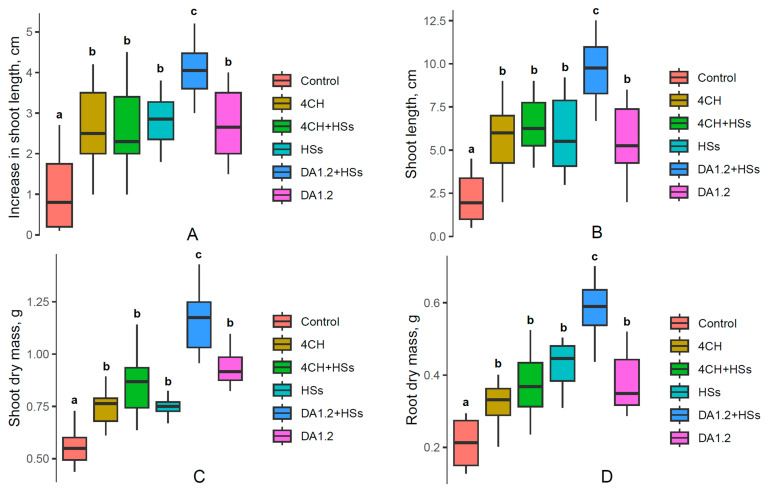
Increase in shoot length during 2 months of growth (**A**), shoot length at the end of the growing season (**B**), and shoot (**C**) and root (**D**) dry mass of one-year-old Scots pine (*Pínus sylvéstris*) seedlings (n = 10) after their treatment with bacterial preparation (*Pseudomonas* sp. (4CH) and *Pseudomonas protegens* (DA1.2)) as well as humic substances (HSs), used separately or combined with each other and without treatment (control) measured at the end of the growing season. Boxplots show medians and distributions of values. The whiskers indicate the minimum and maximum values. Statistically different means are marked with different letters, *p* ≤ 0.05 (one-way ANOVA combined with Duncan’s test).

Measuring the length of shoots of tree seedlings showed that bacterial treatments alone or in combination with humates accelerated shoot elongation in large-leaved linden (Figure 1), horse chestnut (Figure 2), and pine (Figure 3) plants.

Treatment of linden seedlings with individually applied *Pseudomonas protegens* DA1.2 or humates increased the rate of shoot elongation as well as its length and the weight of above- and below-ground organs compared to the control at the end of the growing season (Figure 1), while *Pseudomonas* 4CH produced no effect on the growth of linden seedlings. The addition of *Pseudomonas protegens* DA1.2 increased growth-stimulating effect of humates, while *Pseudomonas* 4CH did not change it. The effect of the treatments was surprisingly pronounced when analyzing root mass (Figure 1D). Root mass was 10 times greater than in the control in the case of a combination of *Pseudomonas protegens* DA1.2 with humates.

Unlike large-leaved linden, the rate of shoot elongation and its mass in horse chestnut showed the highest values when treated with *Pseudomonas* 4CH; the growth-stimulating effect of *Pseudomonas protegens* DA1.2 was lower. Humates themselves accelerated shoot growth, while their addition to *Pseudomonas protegens* DA1.2 did not change the growth-stimulating effect of the strain and decreased the effect of *Pseudomonas* 4CH. The level of the stimulating effect of bacteria on the accumulation of root mass was the same for both strains of bacteria (Figure 2D). The humate preparation showed the least capacity to stimulate root growth, and its addition to bacteria did not change the level of their growth-stimulating effect.

All treatments increased the length of shoots and weight of roots and shoots of Scots pine (*Pínus sylvéstris*) seedlings (Figure 3). The combination of *Pseudomonas protegens* DA1.2 with humates showed the greatest effectiveness in influencing the growth of this plant species.

None of the treatment methods had a stimulating effect on the growth of poplar, mountain ash, or red oak plants (Table 1).

### 2.2. Chlorophyll Content and Nitrogen Balance Index

**Figure 4:**
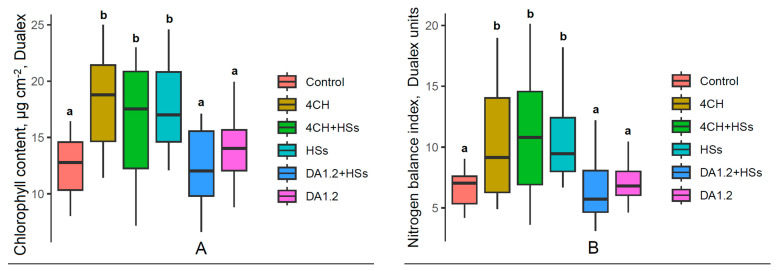
Chlorophyll content (**A**) and nitrogen balance index (**B**) of horse chestnut (*Aesculus hippocastanum*) seedlings one month after the second treatment with a bacterial preparation (*Pseudomonas* sp. (4CH) and *Pseudomonas protegens* (DA1.2)) as well as humic substances (HSs), used separately or combined with each other and without treatment (control). Boxplots show the medians and distributions of values. The whiskers indicate the minimum and maximum values. Statistically different means (n = 20) are marked with different letters, *p* ≤ 0.05 (ANOVA followed by Duncan’s test).

The treatment of horse chestnut (*Aesculus hippocastanum*) seedlings with a humate preparation, bacteria of the *Pseudomonas* sp. strain 4CH and their combination increased chlorophyll content and nitrogen balance index of plants (Figure 4). In all other cases, according to these indicators, chestnut plants and seedlings of other species with all treatments were at the control level (Table 2).

### 2.3. Content of Carbon and Nitrogen

**Figure 5:**
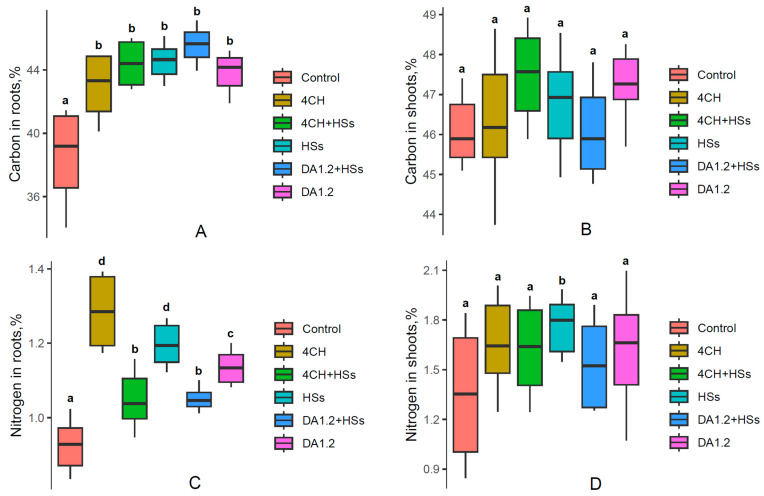
The percentage of carbon (**A**,**B**) and nitrogen (**C**,**D**) in the tissues of shoots (**A**,**C**) and roots (**B**,**D**) of one-year-old Scots pine (*Pínus sylvéstris*) seedlings treated with a bacterial preparation (*Pseudomonas* sp. (4CH) and *Pseudomonas protegens* (DA1.2)) as well as humic substances (HSs), used separately or combined with each other and without treatment (control) at the end of the growing season. Boxplots show the medians and distributions of values. The whiskers indicate the minimum and maximum values. Statistically different means (n = 10) are marked with different letters, *p* ≤ 0.05 (one-way ANOVA combined with Duncan’s test).

The percentage of carbon and nitrogen in pine shoots and roots ranged from 39% to 48% and from 0.9% to 1.8%, respectively, and was higher in shoots than in roots. Treatments increased the carbon content in roots; the increment ranged from 4% to 7% compared to the control (Figure 5). Furthermore, under the influence of all treatments, a small but statistically significant increase in the level of nitrogen was detected in the roots, while in the shoots it was at the control level.

**Figure 6:**
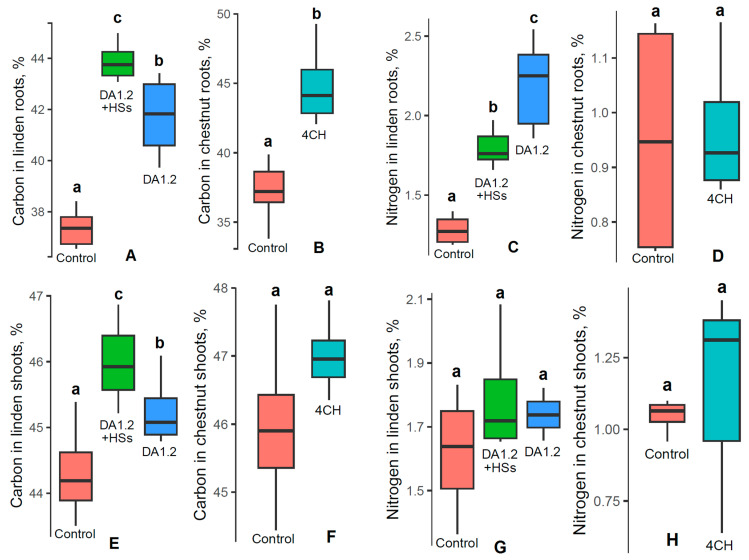
Percentages of carbon (**A**,**B**,**E**,**F**) and nitrogen (**C**,**D**,**G**,**H**) in the tissues of shoots (**E**,**F**,**G**,**H**) and roots (**A**–**D**) of 2-year-old large-leaved linden (*Tília platyphýllos*) (**A**,**C**,**E**,**G**) and horse chestnut (*Aesculus hippocastanum*) (**B**,**D**,**F**,**H**) seedlings treated with a bacterial preparation (*Pseudomonas* sp. (4CH) and *Pseudomonas protegens* (DA1.2)) as well as humic substances (HSs), used separately or combined with each other and without treatment (control). n = 10 at the end of the growing season. Boxplots show the medians and distributions of values. The whiskers indicate the minimum and maximum values. Statistically different means are marked with different letters, *p* ≤ 0.05 (one-way ANOVA combined with Duncan’s test).

Furthermore, we observed a trend of increased carbon content in the roots of linden plants treated with *Pseudomonas protegens* DA1.2 and a combination of bacteria with humates and in the roots of chestnut plants treated with *Pseudomonas* sp. 4CH (Figure 6). These treatments were selected for analysis since they resulted in a significant increase in plant growth (Figure 1 and Figure 2). In the shoots of these plants, the carbon content increased due to treatments only in the linden plants. The nitrogen level was increased by the treatment of linden plants, while in all other variants this indicator was at the control level.

### 2.4. Morphology of Roots of Pine Seedlings

**Figure 7:**
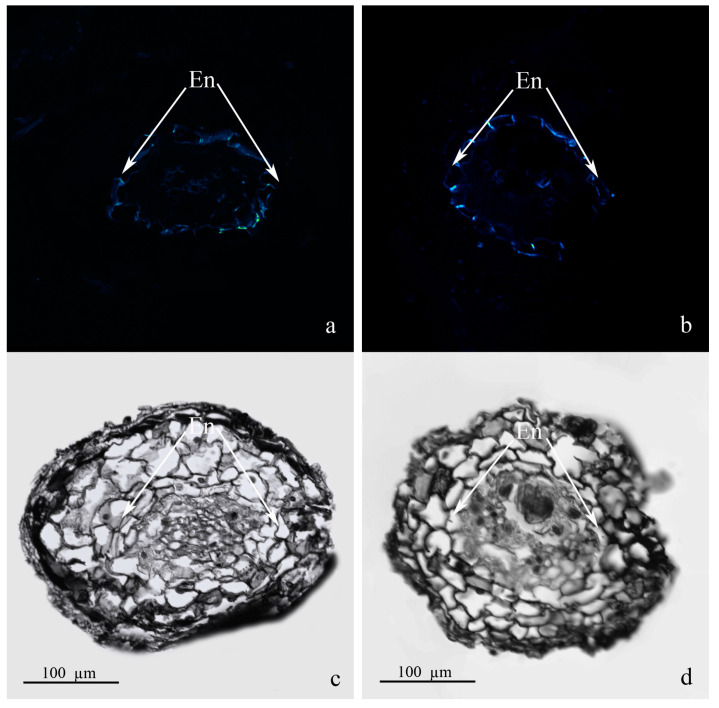
Cross-section of the roots of pine seedlings treated with bacteria of the *Pseudomonas protegens* DA1.2 strain in combination with humates (**a**,**c**) and control (**b**,**d**). Berberine-stained sections (**a**,**b**). Fluorescence was recorded with a confocal microscope and presented in the form of a heat map: green indicates a weaker fluorescence; blue indicates a stronger fluorescence; (**c**,**d**) image in transmitted light. En, endodermis.

The structure of the roots of pine plants was analyzed for the treatment that caused the most noticeable change in the carbon content in the roots (treatment with *Pseudomonas protegens* DA1.2 in combination with humates) (Figure 7). No difference from the structure of the roots of control plants was detected in the roots of treated plants. Berberine fluorescence was observed in the radial walls of endodermis, indicating the presence of lignin characteristic of Casparian bands.

## 3. Discussion

The use of biologically active molecules such as humates and their combination with bacteria had a beneficial effect on the growth of chestnut, large-leaved linden, and pine seedlings, stimulating elongation of their shoots and accumulation of biomass. Humates had a growth-stimulating effect on all three of these plant species. These results of our research are in accordance with the literature on the capacity of humates to stimulate the growth of plants [21]. Most reports about the action of humates were obtained on herbaceous plants [13], but there is also information on their effect on woody plants [3,16,22].

In our experiments, bacteria themselves also stimulated the growth of seedlings. The ability of rhizosphere bacteria to stimulate plant growth is well known, intensively studied, and increasingly used in agriculture [23]. However, information on the impact of bacteria on woody plants is sparse [19,24]. Our experiments demonstrated species specificity of the action of bacterial preparations. *Pseudomonas protegens* DA1.2 had a stronger growth-stimulating effect on linden and pine plants, while *Pseudomonas* sp. 4CH increased the growth rate of chestnut seedlings and did not affect the growth of linden plants. The stimulating effect of *Pseudomonas* sp. 4CH combined with humates on linden was the same as that of humate itself, which indicates a low contribution of bacteria of this strain to the action of humates on linden plants.

In previous studies, the effect of these treatments on normal pine seedlings was investigated [9]. In the present study, we tested their effectiveness on undersized seedlings of pine and confirmed the ability of the treatments to accelerate their growth. According to the standards of Russian pine nurseries, a seedling of this age must be at least 8 mm long. Small seedlings were selected for the present experiments and, if left untreated, they remained stunted by the end of the growing season and were discarded. At the same time, pine seedlings treated with *Pseudomonas protegens* DA1.2 in combination with humates reached the required shoot length in accordance with local standards. 

It is interesting that bacteria not only increased the dry mass of the shoots and roots of plants, but also increased the percentage of carbon in the biomass of their roots. We analyzed sections of plant roots to check the possible effect of increased carbon on their structure. A comparison of the roots of treated and control plants did not reveal any deviations from the norm. It is believed that carbon makes up about 50% of the dry mass of plants; many studies have calculated the carbon content of plants from this ratio [25]. Nevertheless, the increase in carbon content in plant biomass that we found is important because improving the quality of planting material for reforestation is considered in the context of addressing the problem of carbon sequestration. In the future, it is important to evaluate the effect of bacteria on carbon sequestration in older woody plants.

The elemental analysis results also revealed a small but statistically significant increase in nitrogen levels in the roots of treated plants compared to the control. These results can be associated with the ability of the studied bacterial strains to fix nitrogen [9], which increases its availability for plants. Humates also increase the availability of mineral nutrients for plants [22].

Measurement of the nitrogen balance index in chestnut plants also revealed its increase under the influence of some treatments, which confirms their positive effect on the supply of nitrogen to plants. In chestnut plants, an increase in chlorophyll content in the leaves was also recorded, which is not surprising because chlorophyll partially consists of nitrogen. Increased chlorophyll levels apparently contributed to the activation of photosynthesis and plant growth. However, increased chlorophyll levels were not found in all species, in which treatments activated plant growth, and it appears that in these cases, the growth activation was associated with other mechanisms.

As shown above, the bacterial strains used in the present study are capable of synthesizing auxins [9], and humates also increase the level of auxins in plants [15]. This information, as well as the known ability of auxins to stimulate the growth of both the shoots and roots of plants [26], allows us to link the growth-stimulating effect of the treatments with their potential ability to influence the level of auxins in plants.

The additive effect of humates and *Pseudomonas protegens* DA1.2 was manifested in their influence on the accumulation of biomass of the shoots and roots of pine and linden plants. The additive influence of humates and bacteria on cultivated plants may be due to the effect of humates on bacterial growth. We have recently shown that humates increase bacterial abundance in the rhizosphere of wheat [27]. A recent review highlighted the lack of information on the impact of humates on the composition and function of soil microbial communities [28]. Thus, this should be a goal of future research.

## 4. Materials and Methods

### 4.1. Extraction of Humic Substances

Humic substances were extracted from brown coal obtained in the Orenburg region (Russian Federation), with 0.1 M KOH taken in a ratio of 1:10; the mixture was stirred for 2 h at 1500 rpm, and the sediment was removed by centrifugation. After the extraction of humates from brown coal was completed, water was removed under vacuum at a temperature of 55–60 °C to obtain dry humates.

### 4.2. Bacterial Strains and Media for Their Cultivation

Bacterial strains *Pseudomonas protegens* DA1.2 and *Pseudomonas* sp. 4CH [9] were selected from a collection of microorganisms (Ufa Institute of Biology, Ufa, Russia), since their combination with humates was previously shown to stimulate the growth of wheat plants [27]. *Pseudomonas protegens* DA1.2 synthesizes indolylacetic acid, promotes the mobilization of phosphorus from insoluble compounds, fixes atmospheric nitrogen, and exhibits antagonism to microscopic fungi [29]. We cultivated bacteria as described [9]. The number of cells in the cultures was expressed as the number of colony-forming units (CFUs). To obtain a solution for plant treatment, the bacterial suspension was diluted with sterile water.

### 4.3. Studied Species of Tree and Shrub Plants

*Populus italica pyralis* × *P. nigra* is a hybrid obtained at the Bashkir forest experimental station. *Populus* species are often used for reforestation and urban greening; therefore, they were chosen in the present experiments.

*Pinus sylvestris* L. (Scots pine) trees are widespread in Eurasia, from Spain and Great Britain and further east to the middle Amur River in Eastern Siberia. In the north, Scots pine grows all the way to Lapland; further south, it is found in Mongolia and China. The tree is 25–40 m high. The seedlings were obtained from the nursery of the Bashkir State Agrarian University.

Seedlings of the remaining four taxa of trees and shrubs were obtained from the seeds of the collection of the South Ural Botanical Garden-Institute (SUBGI).

*Aesculus hippocastanum* L. (horse chestnut) is native to the Balkans and has been introduced and planted around the world. In the conditions of the South Ural Garden-Institute, it blooms and bears fruit. Horse chestnut trees can grow up to 36 m high, with a low-hanging, spreading, and wide oval dome-shaped crown.

*Sorbus aucuparia* L. (rowan) is native to northern Europe. The tree is 15–20 m tall, usually multi-stemmed.

*Tilia platyphyllos* Scop. (large-leaved linden) is native to much of Europe and is widely grown in parks and city streets. In nature, these trees can grow up to 30–40 m, with a dense, wide-pyramidal crown.

*Quercus rubra* L. (red oak) is naturally distributed across areas of North America. It is a deciduous tree that grows up to 30–40 m in height, with a broadly ovate crown.

### 4.4. Plant Growing Conditions

The experiments were carried out on the territory of the SUBGI in 2023. The climate of the research area (Ufa city) is continental, with long, cold winters and moderately warm and sometimes hot summers. During the year of research, the average temperature from May to September was about 17 °C, and the total precipitation (for these 5 months) was about 150 mm.

Poplar and pine plants grew in soil (gray forest, highly compacted soil). The pH of the soil environment was slightly acidic or close to neutral.

For the experiments, undersized annual seedlings of *Pinus sylvestris* L. were specially selected from the nursery of the Bashkir Agrarian University. Before planting in May, pine seedlings were soaked in 2 L of water with bacterial suspension (10^8^ CFU mL^−1^) and HSs (0.05%) added separately or in combination. The lengths of the main shoots were measured; the seedlings were placed in rows at a distance of 50 cm from each other and treated with 200 mL of preparations of humates (0.02%) and bacteria (10^8^ CFU mL^−1^). Although the humate preparation was alkaline, the soil pH during treatment increased only slightly (from 6.5–6.8 to 6.6–6.9) due to the strong dilution of humates and the buffering capacity of the soil. The distribution of treatment variants between rows was randomized. The seedlings were treated monthly with 200 mL of the bacterial preparations and HS per plant in the same concentrations as during planting. Control plants were treated with equal amounts of water without any additives. In addition, the plants were watered 3 times a week. The growth rate of pine was assessed by measuring the elongation of shoots. At the end of the growing season, the final lengths of the shoots, as well as the dry mass of above- and below-ground organs, were measured. After washing the roots with water, the shoots and roots were dried in an oven at 60 °C until their mass reached a constant value.

Poplar branches were cut, stratified, and, after rooting, planted, as described [9]. Before planting, the roots of the seedlings were soaked in a suspension of bacteria, humates, or a combination of both, as described above. The growth rate of poplar was assessed by shoot elongation over two months.

Seedlings of other trees and shrubs were grown in pots. For sowing, we used seeds from plants of the SUBGI collection obtained through the INDEX SEMINUM seed exchange program. The winter sowing method was used. In late autumn, when the temperature reached about 0 °C, freshly harvested seeds were sown in boxes and left in open ground. With this method of sowing, the seeds undergo natural stratification and germinate in the spring the next year after sowing at their optimal time. Seedlings aged 2–3 years were planted in containers (container volume: large-leaved linden (two years old)—five liters; chestnut (two years old) and rowan (three years old)—3 L; red oak (two years old)—1 L).

To grow seedlings, a universal soil mixture was used: earth, sand, and humus, in a ratio of 3:1:1. Watering was carried out 3 times a week during the growing season. Weeds were removed if necessary. The location of the seedlings in the nursery was sunny, without shading. In mid-May, each plant was treated with 200 mL of bacterial preparation (2 × 10^8^ CFU/mL) and humates (0.02%) applied individually or in mixtures. The next month, the treatment was repeated. The initial lengths of the plant shoots were measured, and shoot elongation was determined 2 months after the first measurements. At the end of the growing season, the shoot lengths, as well as the dry mass of above- and below-ground plant parts, were measured as described above. 

### 4.5. Analysis of Pigment Content

The content of chlorophyll and the nitrogen balance index (NBI) [30] were measured in the 20 biggest leaves of 10 plants (2 leaves from each plant) with a DUALEX SCIENTIFIC+ instrument (FORCE-A, Paris, France) 1 month after the second bacterial treatment, according to the manufacturer’s recommendations.

### 4.6. Determination of C and N

C and N were determined with a EURO EA 3100 elemental analyzer. The percentages of carbon and nitrogen were determined in shoot and root samples, dried as described above, and ground in a mortar with liquid nitrogen. Sampling and introduction into the reactor for combustion was performed as described [31]. The separation of N_2_ and CO_2_ occurred in a built-in gas chromatographic system; detection was carried out with a highly sensitive thermal conductivity detector. Calculations were performed using a special 5.4 Weaver software package.

### 4.7. Preparation and Staining of Root Sections

Fine roots of *Pinus silvestris* (about 0.3 mm in diameter) were fixed in a mixture of formaldehyde, acetic acid, and 70% ethanol (7:7:100). After dehydrating the samples in a series of increasing concentrations of ethyl alcohol, a mixture of ethanol and xylene, and xylene, they were embedded in paraffin. Xylene was evaporated in a thermostat. Paraffin cross-sections of roots were prepared on an MS-2 microtome (RusKhimSnab, Moscow, Russia).

The preparation of paraffin sections for the identification of lignin and suberin in roots was carried out as described previously [32]. Lignin and suberin were identified in accordance with a prior study [33]. For this purpose, after deparaffinization, sections were stained with a 0.1% aqueous solution of berberine hemisulfate (*w*/*v*) for 1 h and then washed with water and additionally stained with toluidine blue to increase the fluorescence intensity. Sections were embedded in a mixture of 0.1% FeCl_3_/50% glycerol and viewed with a Fluoview FV3000 (FV31-HSD) confocal laser scanning microscope (Olympus, Tokyo, Japan): berberine fluorescence was excited by a solid-state laser at 488 nm, and fluorescence emission was detected at 520 nm. 

### 4.8. Statistics

To discriminate means, one-way analysis of variance (ANOVA) with Duncan’s multiple range tests were used (*p* ≤ 0.05), performed in Statistica version 10 (Statsoft, Moscow, Russia). Visualization was performed using the ImageGP online tool (https://doi.org/10.1002/imt2.5, accessed on 2 March 2021).

## 5. Conclusions

An assessment of the combined influences of humates and bacteria on the productivity of agricultural plants showed their greater effectiveness compared to the use of each of them separately [27,34]. To the best of our knowledge, the effect of a combination of humates and bacteria on woody plants was previously carried out only on pine and poplar seedlings [9]. This prior study demonstrated the additive effect of a combination of bacteria and humates on pine seedlings. In the present study, this pattern was revealed on a larger number of plant species (Scots pine, large-leaved linden, and horse chestnut seedlings). However, the applied treatments did not exhibit a growth-stimulating effect on all studied species. Plants of poplar, rowan, and red oak turned out to be unresponsive to treatments. Our results of studying poplar plants confirmed the previously obtained data [9]. At the same time, the fact that one strain of bacteria showed a growth-stimulating effect on some plant species and had a different influence on others indicates the need for selecting the necessary bacteria for each species of woody plant, to exert the appropriate growth-stimulating activity toward each. To date, information has been accumulated on the growth-stimulating effect of a huge number of rhizosphere bacteria (*Bacillus*, *Enterobacter*, *Klebsiella*, *Azobacter*, *Variovorax*, *Azosprillum*, *Serratia*, *Microbacterium*, and others), among which bacteria have be found that can stimulate the growth of woody plants in combination with humates; this can lead to an improved quality of tree planting material for reforestation and increased carbon sequestration. Our results indicate promising research in this direction.

## Figures and Tables

**Table 1:** Elongation of poplar, rowan, and red oak seedling shoots over 2 months when treated with humates and bacterial preparations.

Plant Species	Treatments	Shoot Elongation, cm
Poplar (*Populus italica pyralis × P. nigra*)	Control	13 ± 2
	*Pseudomonas* sp. 4CH	12 ± 1
	*Pseudomonas* sp. 4CH+ humates	12 ± 1
	Humates	11 ± 3
	*Pseudomonas protegens* DA1.2+humates	12 ± 1
	*Pseudomonas protegens* DA1.2	11 ± 2
Rowan (*Sorbus aucuparia*)	Control	27 ± 5
	*Pseudomonas* sp. 4CH	26 ± 4
	*Pseudomonas* sp. 4CH+ humates	25 ± 4
	Humates	23 ± 4
	*Pseudomonas protegens* DA1.2+humates	24 ± 3
	*Pseudomonas protegens* DA1.2	23 ± 2
Red oak (*Quercus rubra*)	Control	9.8 ± 1.7
	*Pseudomonas* sp. 4CH	9.2 ± 1.4
	*Pseudomonas* sp. 4CH+ humates	7.5 ± 2
	Humates	7.4 ± 2.2
	*Pseudomonas protegens* DA1.2+humates	8.5 ± 1.7
	*Pseudomonas protegens* DA1.2	7.9 ± 1.2

**Table 2:** Chlorophyll content and nitrogen balance index of plants treated with bacteria and humates individually and in combination.

Plant Species	Treatments	Chlorophyll Content, µg cm^−2^, Dualex	Nitrogen Balance Index, Dualex Units
Poplar (*Populus italica pyralis × P. nigra*)	Control	21.5 ± 0.9	10.9 ± 0.6
	*Pseudomonas* sp. 4CH	21.0 ± 1.1	10.4 ± 0.6
	*Pseudomonas* sp. 4CH+ humates	24.7 ± 2.2	12.9 ± 1.1
	Humates	21.0 ± 1.4	10.3 ± 0.7
	*Pseudomonas protegens* DA1.2+humates	23.2 ± 1.2	11.5 ± 0.6
	*Pseudomonas protegens* DA1.2	19.7 ± 1.8	10.3 ± 1.0
Rowan (*Sorbus aucuparia*)	Control	13.4 ± 0.6	6.5 ± 0.2
	*Pseudomonas* sp. 4CH	13.8 ± 0.8	7.4 ± 0.6
	*Pseudomonas* sp. 4CH+ humates	14.8 ± 0.5	8.8 ± 1.2
	Humates	13.5 ± 0.7	8.0 ± 0.8
	*Pseudomonas protegens* DA1.2+humates	15.1 ± 0.9	7.7 ± 0.8
	*Pseudomonas protegens* DA1.2	15.0 ± 0.9	7.0 ± 0.6
Red oak (*Quercus rubra*)	Control	14.6 ± 0.9	10.5 ± 0.7
	*Pseudomonas* sp. 4CH	16.3 ± 0.8	13.1 ± 1.4
	*Pseudomonas* sp. 4CH+ humates	14.8 ± 0.7	11.2 ± 0.8
	Humates	15.0 ± 1.0	13.0 ± 1.2
	*Pseudomonas protegens* DA1.2+humates	15.1 ± 1.1	12.9 ± 1.4
	*Pseudomonas protegens* DA1.2	15.1 ± 0.7	11.4 ± 0.7

## Data Availability

Data are contained within the article.

## References

[1] Rouphael Y., Colla G. (2018). Synergistic biostimulatory action: Designing the next generation of plant biostimulants for sustainable agriculture. Front. Plant Sci..

[2] Nardi S., Ertani A., Francioso O. (2016). Soil-root cross-talking: The role of humic substances. J. Plant Nutr. Soil Sci..

[3] Hriciková S., Kožárová I., Hudáková N., Reitznerová A., Nagy J., Marcinčák S. (2023). Humic substances as a versatile intermediary. Life.

[4] Lind E., Prade T., Sjoman Deak J., Levinsson A., Sjöman H. (2023). How green is an urban tree? The impact of species selection in reducing the carbon footprint of park trees in Swedish cities. Front. Sustain. Cities.

[5] Park H.-M., Jo H.-K., Kim J.-Y. (2021). Carbon footprint of landscape tree production in Korea. Sustainability.

[6] Waring B., Neumann M., Prentice I.C., Adams M., Smith P., Siegert M. (2020). Forests and decarbonization—Roles of natural and planted. Front. For. Glob. Change.

[7] Fargione J., Haase D.L., Burney O.T., Kildisheva O.A., Edge G., Cook-Patton S.C., Chapman T., Rempel A., Hurteau M.D., Davis K.T. (2021). Challenges to the reforestation pipeline in the United States. Front. For. Glob. Change.

[8] Pinto J.R., Sloan J.L., Ervan G., Burney O.T. (2023). Physiological and morphological responses of *Pinus ponderosa* seedlings to moisture limitations in the nursery and their implications for restoration. Front. Plant Sci..

[9] Nazarov A., Chetverikov S., Chetverikova D., Tuktarova I., Ivanov R., Urazgildin R., Garankov I., Kudoyarova G. (2023). Microbial preparations combined with humic substances improve the quality of tree planting material needed for reforestation to increase carbon sequestration. Sustainability.

[10] Vejan P., Abdullah R., Khadiran T., Ismail S., Nasrulhaq Boyce A. (2016). Role of plant growth promoting rhizobacteria in agricultural sustainability—A review. Molecules.

[11] Abdel Latef A.A.H., Omer A.M., Badawy A.A., Osman M.S., Ragaey M.M. (2021). Strategy of salt tolerance and interactive impact of *Azotobacter chroococcum* and/or *Alcaligenes faecalis* inoculation on canola (*Brassica napus* L.) plants grown saline soil. Plants.

[12] Backer R., Rokem J.S., Ilangumaran G., Lamont J., Praslickova D., Ricci E., Smith D.L. (2018). Plant growth-promoting rhizobacteria: Context, mechanisms of action, and roadmap to commercialization of biostimulants for sustainable agriculture. Front. Plant Sci..

[13] Canellas L.P., Olivares F.L., Aguiar N.O., Jones D.L., Nebbioso A., Mazzei P. (2015). Humic and fulvic acids as biostimulants in horticulture. Sci. Hortic..

[14] Olaetxea M., de Hita D., Garcia C.A., Fuentes M., Baigorri R., Mora V., Garnica M., Urrutia O., Erro J., Zamarreño A.M. (2018). Hypothetical framework integrating the main mechanisms involved in the promoting action of rhizospheric humic substances on plant root and shoot-growth. Appl. Soil Ecol..

[15] Nazarov A.M., Garankov I.N., Tuktarova I.O., Salmanova E.R., Arkhipova T.N., Ivanov I.I., Feoktistova A.V., Prostyakova Z.G., Kudoyarova G.R. (2020). Hormone balance and shoot growth in wheat (*Triticum durum* Desf.) plants as influenced by sodium humates of the granulated organic fertilizer. Sel’skokhozyaistvennaya Biol..

[16] Alva A.K., Obreza T.A. (1998). By-product iron-humate increases tree growth and fruit production of orange and grapefruit. HortScience.

[17] Cahyo A.N., Ardika R., Saputra J., Wijaya T. (2014). Acceleration on the growth of rubber planting materials by using foliar application of humic acid. J. Agric. Sci..

[18] Chaiya L., Gavinlertvatana P., Teaumroong N., Pathom-aree W., Chaiyasen A., Sungthong R., Lumyong S. (2021). Enhancing Teak (*Tectona grandis*) seedling growth by rhizosphere microbes: A sustainable way to optimize agroforestry. Microorganisms.

[19] Shinde S., Cumming J.R., Collart F.R., Noirot P.H., Larsen P.E. (2017). *Pseudomonas fluorescens* transportome is linked to strain-specific plant growth promotion in aspen seedlings under nutrient stress. Front. Plant Sci..

[20] Ivetić V., Devetaković J., Nonić M., Stanković D., Šijačić-Nikolić M. (2016). Genetic diversity and forest reproductive material—From seed source selection to planting. iForest.

[21] Calvo P., Nelson L., Kloepper J.W. (2014). Agricultural uses of plant biostimulants. Plant Soil.

[22] Ren H., Islam M.S., Wang H., Guo H., Wang Z., Qi X., Zhang S., Guo J., Wang Q., Li B. (2022). Effect of humic acid on soil physical and chemical properties, microbial community structure, and metabolites of decline diseased bayberry. Int. J. Mol. Sci..

[23] Kudoyarova G., Arkhipova T., Veselov D. (2023). Water relations in plants treated with growth promoting rhizosphere bacteria. Plant Soil.

[24] Lotfi N., Soleimani A., Çakmakçı R., Mohammadi P. (2022). Characterization of plant growth-promoting rhizobacteria (PGPR) in Persian walnut associated with drought stress tolerance. Sci. Rep..

[25] Yin W., Yin M., Zhao L., Yang L. (2012). Research on the measurement of carbon storage in plantation tree trunks based on the carbon storage dynamic analysis method. Int. J. For. Res..

[26] Woodward A.W., Bartel B. (2005). Auxin: Regulation, action, and interaction. Ann. Bot..

[27] Feoktistova A., Timergalin M., Chetverikov S., Nazarov A., Kudoyarova G. (2023). Effects on *Pseudomonas plecoglossicida* 2,4-D and humic substances on the growth, pigment indices and concentration of hormones in wheat seedlings grown under water deficit. Microorganisms.

[28] Lumactud R.A., Gorim L.Y., Thilakarathna M.S. (2022). Impacts of humic-based products on the microbial community structure and functions toward sustainable agriculture. Front. Sustain. Food Syst..

[29] Chetverikov S.P., Chetverikova D.V., Bakaeva M.D., Kenjieva A.A., Starikov S.N., Sultangazin Z.R. (2021). A promising herbicide-resistant bacterial strain of pseudomonas protegens for stimulation of the growth of agricultural cereal grains. Appl. Biochem. Microbiol..

[30] Zhang K., Liu X., Ma Y., Zhang R., Cao Q., Zhu Y., Cao W., Tian Y. (2019). A comparative assessment of measures of leaf nitrogen in rice using two leaf-clip meters. Sensors.

[31] Galimullin R.R., Sigaeva N.N., Glukhov E.A., Spirikhin L.V., Kolesov S.V. (2019). Radical-initiated (co)polymerization of methacrylates in the presence of organometallic iron complexes. Russ. J. Appl. Chem..

[32] Zaqout S., Becker L.L., Kaindl A.M. (2020). Immunofluorescence staining of paraffin sections step by step. Front. Neuroanat..

[33] Junghans U., Langenfeld-Heyser R., Polle A., Teichmann T. (2004). Effect of auxin transport inhibitors and ethylene on the wood anatomy of poplar. Pl. Biol..

[34] Alharbi K., Rashwan E., Hafez E., Omara A.E.-D., Mohamed H.H., Alshaal T. (2022). Potassium humate and plant growth-promoting microbes jointly mitigate water deficit stress in soybean cultivated in salt-affected soil. Plants.

